# The association of social capital with depression and quality of life in school-aged children

**DOI:** 10.1371/journal.pone.0262103

**Published:** 2022-01-13

**Authors:** Hiroyuki Mori, Michio Takahashi, Masaki Adachi, Hiroki Shinkawa, Tomoya Hirota, Tomoko Nishimura, Kazuhiko Nakamura

**Affiliations:** 1 Research Center for Child Mental Development, Graduate School of Medicine, Hirosaki University, Hirosaki, Aomori, Japan; 2 Department of Neuropsychiatry, Graduate School of Medicine, Hirosaki University, Hirosaki, Aomori, Japan; 3 Department of Clinical Psychological Science, Graduate School of Health Sciences, Hirosaki University, Aomori, Japan; 4 Department of Psychiatry and Behavioral Sciences, Weill Institute for Neurosciences, University of California San Francisco, San Francisco, CA, United States of America; 5 Research Center for Child Mental Development, Hamamatsu University School of Medicine, Hamamatsu, Shizuoka, Japan; Shinshu University School of Medicine, JAPAN

## Abstract

Social capital is an important factor that affects mental health. The purpose of this study was to examine the relationship between social capital and depression and between social capital and quality of life (QoL) in children in elementary and junior high school and to examine how this relationship differs in relevant patterns at both the individual- and school-level. The study was conducted in all elementary and junior high schools in a single municipality; the subjects consisted of 3,722 elementary school and 3,987 junior high school students (aged from 9 to 15). A multilevel linear mixed effect model analysis revealed that all three subscales of social capital were associated with depression and QoL at the individual-level: The school social capital at the individual-level showed the strongest association with depression and QoL. We also found that some of social capital at the school-level was associated with depression and QoL. An interactive effect was observed between educational stage (elementary and junior high) and some of social capital subscales. Specifically, the inverse association between school social capital and depression was stronger among the junior high students, while the positive association between school and neighborhood social capital and QoL was stronger among the elementary students. These interactions suggest that social capital impacts depression and QoL differently in elementary and junior high students. These findings suggest that the degree of association of social capital domains differs in mental health among the educational stage.

## Introduction

According to the World Health Organization, 10–20% of adolescents experience psychiatric disturbance or mental health problems, and half of all such conditions emerge by the age of 14 [1]. Mental health problems in young people can diminish quality of life (QoL), exacerbate problem behavior, and reduce school attendance [2]. They can also pose a major risk for mental health decline in adulthood [3, 4]. In Japan, school absenteeism has been rising in recent years [5], and suicides among young people reached a record high in 2020: mortality rates were 7.0 among 19-year-olds or younger [6]. Given multiple reports highlighting depression as a risk factor for school absenteeism and suicide [7, 8], the prevention of mental health problems in children and adolescents is essential in Japan and other countries.

The risk factors for major depressive disorder in adolescence include not only individual risk factors, such as emotion-regulation capacities and coping mechanisms, but also contextual factors such as school and relationships with family and friends [9].

Social capital encompasses these contextual factors and widely debated concepts based on the work of Bourdieu [10], Coleman [11], and Putnam [12, 13]. Two main approaches exist with regard to the measurement of social capital, which are generally referred to as social cohesion and the network-based views [14, 15]. Social cohesion perspectives, represented by Coleman [11] and Putnam [12, 13], emphasize trust (in general and in particular others) and formal participation in civic associations. Network perspectives, based on the work by Bourdieu [10] and Lin [16], emphasize informal social connections of individuals and the diversity of resources accessed through those connections [17, 18]. For example, in terms of network perspectives, social networks and social resouces assesed by position generator, resource generator, name generator, and the ISSP 2017 ’social networks and social resources’ [19]. Villalonga-Olives and Kawachi [14] proposed that the definition of social capital is conceptualized as 1) a group attribute, that are the resources (e.g. trust, norms, and the exercise of sanctions) available to members of social groups, and 2) an individual attribute as well as a property of the collective which is the resources (e.g. social support, information channels, and social credentials) that are embedded within an individual’s social networks. Furthermore, Kawachi and Berkman [20] emphasize that social capital is inherent in the structure of social relationships (i. e. social capital is considered to be an ecological system). In fact, social capital, including individual- and ecological-level, is associated with a various of health outcomes [21].

Social capital has garnered attention for its association with societal problems such as delinquency, crime, suicide mortality, and bullying. For example, a study in 11 European countries reported an inverse relationship between social capital and national suicide rates and concluded that social capital may protect against suicide at the national level [22]. Similarly, a study in the US reported that lack of social trust was associated with crime [23].

Several categories of social capital have been previously examined. Some categories are based on the components of social capital, which include “structural social capital” and “cognitive social capital.” Structural social capital describes formal and informal networks, social organizations, and social activities involving roles or norms, while cognitive social capital describes trust and reciprocity [24, 25]. Other categories, based on the population of interest, include “community/neighborhood social capital” (civic activities, trust, safety, neighborhood environment) and “family social capital” (family composition, cohesion) [26].

In recent years, there has been an increase in research on social capital for children and adolescents (e.g. [27, 28]), but several problems have been pointed out. First, when it comes to children and adolescents, the literature highlights the need to consider “school social capital” on the grounds that children and adolescents spend most of their day in school, and because school serves as a key social environment for their development [29–31]. School social capital is characterized as a concept that incorporates resources from the social networks found in schools, which include rules and norms regulating peer relationships [32]. Evidence shows that school social capital is a major factor in the mental health of children and adolescents and that it is inversely associated with problem behavior and mental health problems [33–36]. For example, higher school commitment is associated with a lower degree of depression and higher self-esteem [37]; higher perceived safety at school is associated with higher self-esteem [38]. Furthermore, one study reported that mental health problems were associated not only with school social capital at the individual-level but also with school social capital at the school-level [34]. This suggests the importance of considering school social capital at both individual-level and ecological-level. Other reports reveal that both neighborhood social capital and family social capital are positively associated with QoL [33, 39].

Second, although previous research showed that cognitive social capital has a stronger impact on mental health than structural social capital does [40], a critical review revealed that most studies in this area examined only structural social capital including family composition and local residential conditions using macro-statistical data; few studies considered the subjective assessment of cognitive social capital by the young people themselves [41]. Likewise, few studies have focused on school social capital despite evidence that the school environment significantly affects mental health [42].

Another issue concerns the tools for assessing social capital. Reliable and valid assessment tools for adolescents’ social capital are scarce [43], and there are discrepancies in how social capital is operationalized. For example, many of the tools consist of one or two independent questions (measuring trust, connectedness, or the like) [44]. Given the findings from epidemiological studies reporting an increase in the onset of depression in adolescence [3, 45], it is important for understanding and prevention that the association between social capital and their mental health problems, at both the individual—and collective (school) -level is examined further.

Identifying the components of social capital that associate significantly with mental health across two age ranges (elementary/junior high) should provide valuable insight for developing an effective preventive strategy. Several studies have shown that mental health problems increase in adolescence [3, 45], while age differences have been found to affect the association between neighborhood social capital and both mental health and QoL [46]. Research has shown that profiles of social capital, within the context of family and school, and among peers, were different in younger and older adolescents [47]. In Japan and other countries, some aspects of the learning environment differ between elementary and junior high school, such as the size of campus, the number of students, and whether students are taught by multiple subject specialists [48, 49]. Further, the connectedness and relationships of junior high students change qualitatively because these students become more susceptible to peer influence than elementary students [50, 51].

Given the above issues, the purpose of the present study was to clarify the relationship between social capital, including school social capital, and both depression and QoL in school-aged children at both the individual- and school-levels. The present study also sought to verify whether this relationship differs between children in elementary school and those in junior high school because differences in age and school environment may affect the relationship.

## Methods

### Subjects

Data were collected from school cohorts via a survey administered by the Research Center for Child Mental Development in collaboration with the local education committee. The survey was conducted in September 2018 among 3,949 fourth to sixth grade elementary students (equivalent to 9–12 years of age) and 4,235 first to third grade junior high students (equivalent to 12–15 years of age) attending public schools in a single municipality. Hirosaki City has 52 public elementary and junior high schools (35 elementary and 17 junior high schools), and only one private school (junior high school). About 11,925 (99.4%) of children are enrolled in public elementary and junior high schools. A total of 8,184 (68.2%) students were surveyed, and 7,892 (65.8%) responded. Of these, we excluded 183 students (1.5%) who had incomplete responses. Finally, 7,709 (64.3%) students submitted complete responses, and their data were included in the analysis. Of the 7,709 respondents, 3,722 were in elementary school (1,872 male, 1,850 female) and 3,987 in junior high school (2,002 male, 1,985 female).

### Survey procedure, ethical considerations

In accordance with the manual of survey procedures, teachers distributed questionnaire forms to students in the classroom all at once. Prior to the survey, the students’ parents/guardians were informed in writing of the study’s purpose, that they were free to decide whether their child would participate and that their child would suffer no disadvantage if they declined to let the child participate. Parents/guardians were instructed to contact the Research Center for Child Mental Development if they were unwilling to let their child participate. In this circumstance, the center would inform the relevant school to refrain from giving the student a questionnaire. The students themselves received a verbal briefing from their teacher informing them of the study purpose, that they were free to decide whether to participate and that they would suffer no disadvantage for declining to participate. The study was approved by the ethics committee of the Hirosaki University Graduate School of Medicine.

### Instruments

#### Social Capital Questionnaire for Adolescent Students

The SCQ-AS was developed to measure the social capital of adolescent students and includes safety, trust, and cohesion, which have been measured in previous studies [24, 26]. The draft SCQ-AS consisted of 16-items: three subscales, “social network/cohesion/sense of belonging", “trust", "autonomy and control”, including school, family and neighborhood components. As a result of evaluation of psychometric properties, the four items referred to bullying and parental control were excluded [44]. Therefore, the original version of the scale has four subscales: “school cohesion,” “school friendship,” “neighborhood social cohesion,” and “trust: school/neighborhood.” This scale is characterized by containing items related to the school social capital [44]. Compared to other scales for example, the scale developed by Sampson and colleagues [52] can measure neighborhood social capital, but it has been limited to collective efficacy in neighborhoods and residential areas. The social capital measurement tool developed by Takakura and colleagues [53] can measure social capital including trust and reciprocity in schools and neighborhoods. However, it does not include safety and friends items, and has not been validated with early adolescents. This study used a Japanese version of the SCQ-AS, which provides a quantitative measure of social capital, and has a confirmed construct validity [44, 54]. The scale consists of 12 items scored on a 3-point scale. The total score ranges from 12 to 36, with a higher score indicating greater social capital. Only three subscales are used in the Japanese version because our research team previously examined the factor structure of the Japanese-version of SCQ-AS and confirmed that the following three-factor model had best-fit indices: “school trust and social cohesion” (eight items; school social capital), “perceived safety in school and neighborhood” (two items; safety), and “neighborhood trust and social cohesion” (two items; neighborhood social capital) [54].

#### Depression Self-Rating Scale for Children

Depression was measured using a Japanese version of the Depression Self-Rating Scale for Children (DSRS-C) [55, 56]. The DSRS-C consists of 18 items scored on a 3-point scale (never  =  0; sometimes  =  1; always  =  2). The total score ranges from 0 to 36, with a higher score indicating a more severe level of depression. The Japanese version reported good reliability in elementary and middle school students (Cronbach’s alpha of 0.77) [55].

#### Pediatric Quality of Life Inventory 4.0 Generic Core Scales

QoL was measured using a Japanese version of the Pediatric Quality of Life Inventory 4.0 Generic Core Scales (PedsQL) [57, 58]. The PedsQL consists of 23 items scored on a 5-point scale. The instrument has four domains: “physical functioning” (eight items), “emotional functioning” (five items), “social functioning” (five items), and “school functioning” (five items) [57, 58]. The total score ranges from 0 to 100, with a higher score indicating better QoL.

### Statistical analysis

Preliminary data analysis included an examination of the descriptive statistics for all variables. We conducted a multilevel mixed-effects analysis to examine the individual- and school-level effects of social capital on depression and QoL. Multilevel linear mixed-effect modeling was applied to the data, as the method allowed us to examine individual- and school-level effects simultaneously by splitting the variance of the observed variables into the variance components for each level [59]. School-level aggregated scores were calculated for student-reported scores on each of the SCQ-AS subscales and used as the school means. These aggregated scores represent students’ shared perceptions of school-wide social capital. Sex was entered as a confounding variable in view of evidence indicating sex differences in adolescent mental health [60, 61]. Model 0 was an empty model (i.e. the unconditional model) which was used to calculate the intraclass correlation coefficient (ICC) and design effect (DEFF) to determine if multilevel modeling is needed [62]. Sex (male/female) and educational stage (elementary/junior high) were entered in Model 1, in which the association between outcome variables and only individual-level variables were examined. Next, the SCQ-AS subscale score was entered as a main effect in Model 2. As the next step, individual-level interactions (educational stage with SCQ-AS subscale) were entered in Model 3 to determine the interactive effect between the educational stage and each subscale score. All of school-level variables (school size [the number of students per school] and all of SCQ-AS subscales) were added to Model 4. Finally, cross-level interaction variables between the educational stage and school-level aggregated scores for each SCQ-AS subscale were added to Model 5. Since multilevel modeling with cross-level interaction should be included random slope [63], we estimated random slope for individual-level variable. Furthermore, to examine the differences in the association between social capital and response variables by the educational stage, significant interactions were probed with simple slopes analysis.

We utilized group-mean centering by school for the individual-level predictors, grand-mean centering for the school-level predictors as is recommended for research questions where the effects of the individual-level predictors and the corresponding higher-level predictors are compared [64]. Model fit was evaluated using the deviance information criterion (DIC) and ΔDIC > 7 was deemed to fit better [65]. The significance threshold was set at *p* < .05. All analyses were performed using Mplus version 8.6 [66].

## Results

Descriptive statistics for the 3,722 elementary school students and 3,987 junior high school students are shown in Table 1, respectively. The compositional variables show that mean grade of the respondents is 5.03±0.82 grade in elementary and 8.02±0.82 grade in junior high school. Sex was almost equally distributed in both elementary and junior high schools. The school characteristics of 35 elementary and 17 junior high schools are also shown in Table 1. The mean of school size was 106.34±69.94 students and ranged from 4 to 263 students in elementary and 234.53±186.79 students and range from 10 to 602 students in junior high school.

**Table 1:** Descriptive statistics.

	Elementary school			Junior high school		
**Individual-level: Student Characteristics**	*n* = 3722			*n* = 3987		
	*M (SD)*	*Min*	*Max*	*M (SD)*	*Min*	*Max*
Grade	5.03 (0.82)	4.00	6.00	8.03 (0.82)	7.00	9.00
Sex (Male)	50.30%	0.00	1.00	50.20%	0.00	1.00
DSRS-C	7.70 (5.58)	0.00	35.00	8.92 (6.25)	0.00	33.00
PedsQL	82.71 (13.89)	8.70	100.00	88.78 (12.98)	0.00	100.00
SCQ-AS						
Total score	31.06 (4.03)	14.00	36.00	30.93 (4.23)	12.00	36.00
School trust and social cohesion	20.60 (2.92)	8.00	24.00	20.69 (3.04)	8.00	24.00
Perceived safety in school and neighborhood	5.38 (0.94)	2.00	6.00	5.29 (1.01)	2.00	6.00
Neighborhood trust and social cohesion	5.08 (1.05)	2.00	6.00	4.95 (1.09)	2.00	6.00
**School-level: School Characteristics**	*n* = 35			*n* = 17		
	*M (SD)*	*Min*	*Max*	*M (SD)*	*Min*	*Max*
School size	106.34 (69.94)	4.00	263.00	234.53 (186.79)	10.00	602.00
SCQ-AS						
Total score	31.45 (1.38)	29.06	33.53	31.16 (0.78)	30.06	32.52
School trust and social cohesion	20.82 (0.97)	18.83	22.27	20.82 (0.50)	20.08	21.52
Perceived safety in school and neighborhood	5.44 (0.20)	5.05	5.83	5.32 (0.16)	5.08	5.61
Neighborhood trust and social cohesion	5.19 (0.28)	4.69	5.66	5.02 (0.25)	4.60	5.55

DSRS-C, Depression Self-Rating Scale for Children; PedsQL, Pediatric Quality of Life Inventory 4.0 Generic Core Scales; SCQ-AS, Social Capital Questionnaire for Adolescent Students.

**Fig 1:**
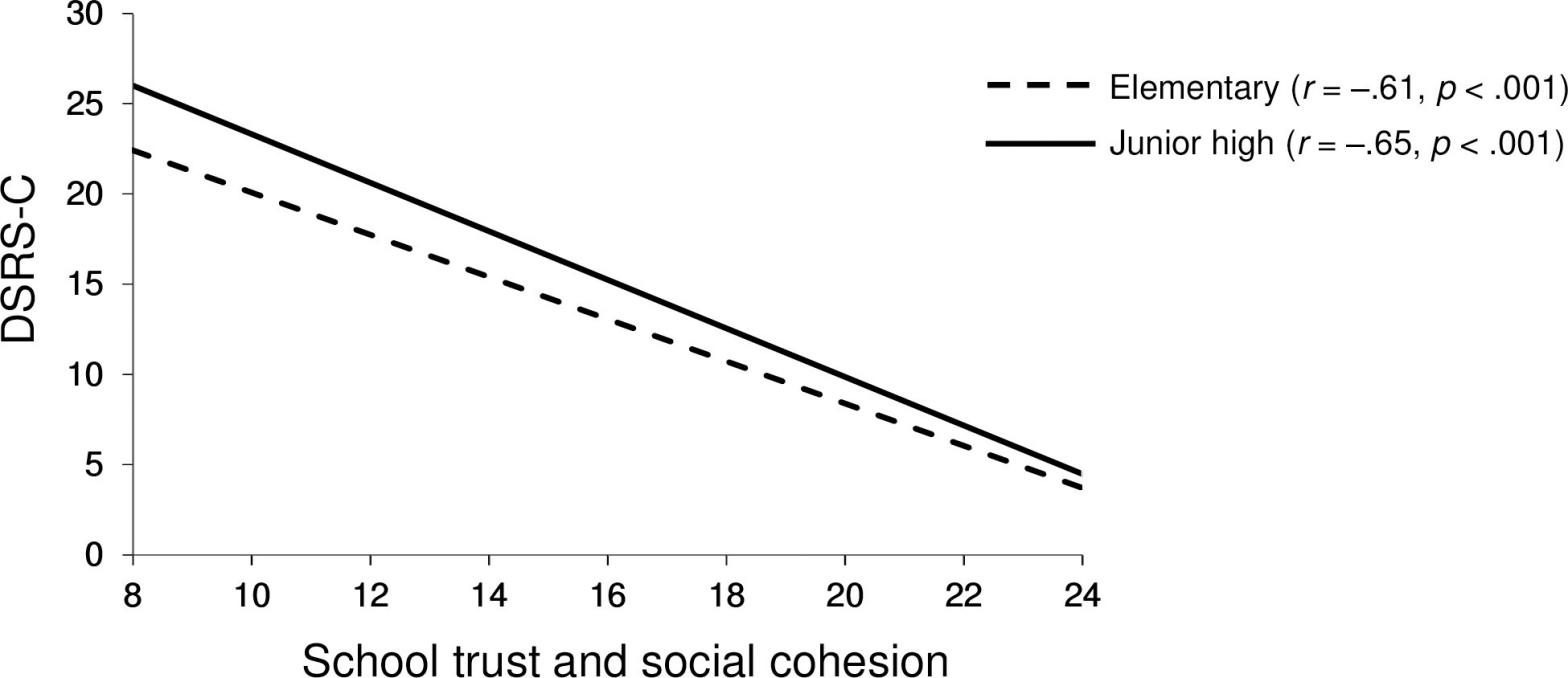
Interaction between school social capital and the educational stage on depression. Plots of the impacts of the interaction between “school trust and cohesion” and educational stage on depression. DSRS-C, Depression Self-Rating Scale for Children.

Table 2 shows the results of the multilevel linear mixed-effect in which the criterion variable was depression, measured by the DSRS-C. Model 0 is an empty model without explanatory variables. The ICC was 0.04, which means that 4% of the observed individual differences in depression can be attributed to the school-level. DEFF was 309.32, which means that this data has hierarchical structure and multilevel modeling is needed. Since DIC decreased with each model, Model 5 was adopted as the final model (individual-level: *R*^2^ = .38, *p* < .001, school-level: *R*^2^ = .79, *p* < .001). As regard with individual-level, the educational stage and all three SCQ-AS subscales exhibited a significant main effect on DSRS-C (depression). The subscale “school trust and social cohesion” (β = –0.47, *p* < .001) showed the stronger relationship with depression than other subscales at individual-level (“perceived safety in school and neighborhood” (β = –0.14, *p* < .001) and “neighborhood trust and social cohesion” (β = –0.09, *p* < .001). The significant interaction of the educational stage with the subscale “school trust and social cohesion” (β = –0.02, *p* < .001) indicated that the relationship between school social capital and depression varied between the educational stage (elementary and junior high). The simple slopes analysis revealed that “school trust and social cohesion” was more strongly associated with depression among junior high students (*B* = –0.98, *p* < .001) than elementary students (*B* = –1.14, *p* < .001) (Fig 1). The higher the score on this subscale, the less severe depression, especially among junior high students.

**Table 2:** Multilevel linear mixed effect model coefficients for DSRS-C.

	Model 0	Model 1	Model 2	Model 3	Model 4	Model 5
	β (*SE*)	β (*SE*)	β (*SE*)	β (*SE*)	β (*SE*)	β (*SE*)
**Individual-level**						
Sex		0.05 (0.02) ***	0.03 (0.01) ***	0.07 (0.01) ***	0.07 (0.01) ***	0.07 (0.01) ***
Educational Stage		0.13 (0.01) ***	0.87 (0.03) ***	0.45 (0.08) ***	0.39 (0.07) ***	4.79 (1.21) ***
SCQ-AS						
School trust and social cohesion			–0.26 (0.02) ***	–0.48 (0.02) ***	–0.49 (0.01) ***	–0.47 (0.01) ***
Perceived safety in school and neighborhood			–0.07 (0.01) ***	–0.14 (0.02) ***	–0.14 (0.01) ***	–0.14 (0.01) ***
Neighborhood trust and social cohesion			–0.05 (0.01) ***	–0.09 (0.02) ***	–0.09 (0.02) ***	–0.09 (0.01) ***
**Individual-level Interaction**						
Educational Stage × School trust and social cohesion				–0.28 (0.07) ***	–0.28 (0.07) ***	–0.02 (0.01) ***
Educational Stage × Perceived safety in school and neighborhood				–0.05 (0.05)	–0.04 (0.05)	–0.00 (0.01)
Educational Stage × Neighborhood trust and social cohesion				0.01 (0.05)	0.01 (0.05)	0.00 (0.01)
**School-level**						
School size					–0.05 (0.09)	–0.07 (0.17)
SCQ-AS						
School trust and social cohesion					–0.59 (0.15) ***	–0.42 (0.14) *
Perceived safety in school and neighborhood					–0.40 (0.14) ***	–0.73 (0.13) ***
Neighborhood trust and social cohesion					–0.05 (0.18)	0.04 (0.18)
**Cross level interaction (Individual × school-level)**						
Educational Stage × School trust and social cohesion						–0.05 (0.29)
Educational Stage × Perceived safety in school and neighborhood						0.55 (0.18) **
Educational Stage × Neighborhood trust and social cohesion						–0.20 (0.22)
**Model Information criteria**						
Deviance (DIC)	49209.81	49192.78	43454.03	44939.31	44921.26	44855.62
**ICC**	0.04	0.03	0.81	0.06	0.01	0.00

** *p* < .01

*** *p* < .001.

DSRS-C, Depression Self-Rating Scale for Children; SCQ-AS, Social Capital Questionnaire for Adolescent Students; DIC, Deviance information criterion.

**Fig 2:**
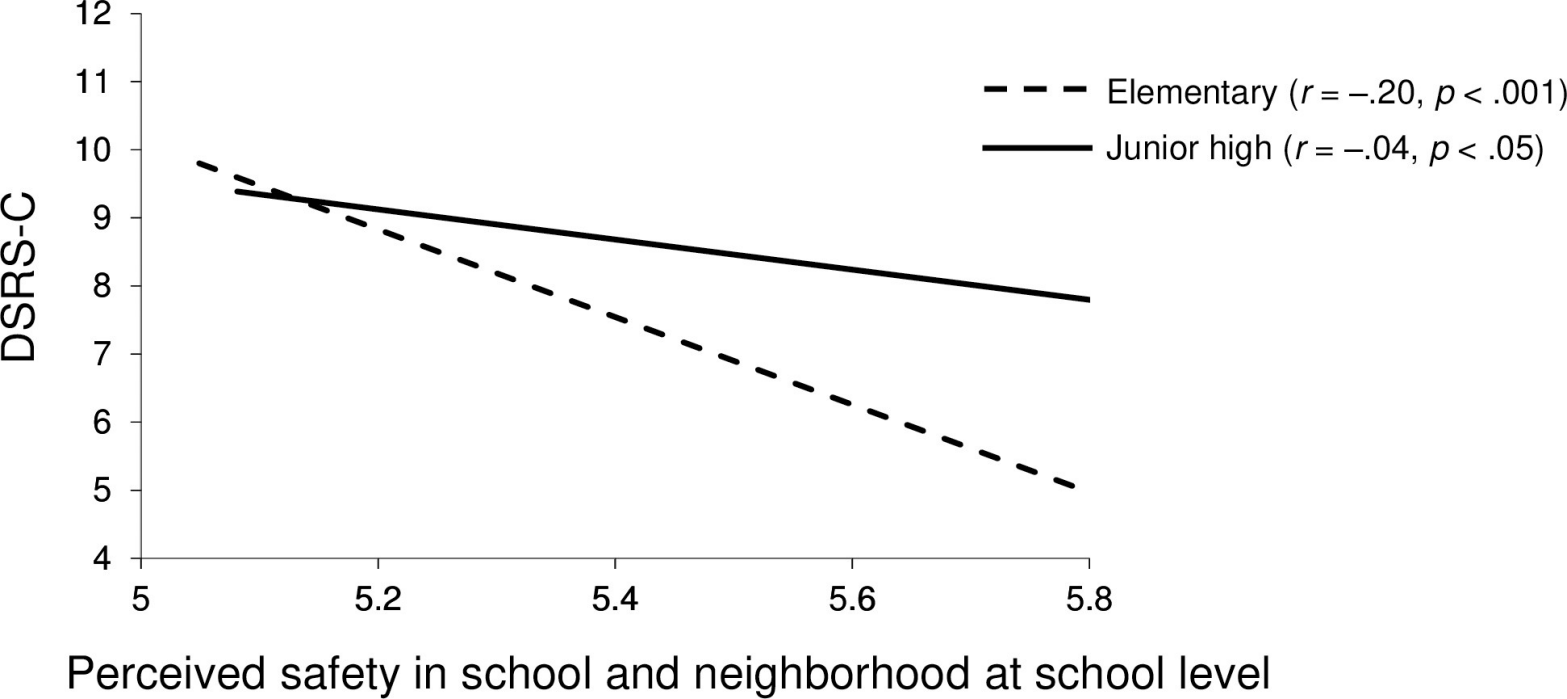
Cross-level interaction between safety and the educational stage on depression. Plots of the impacts of the cross-level interaction between “perceived safety in school and neighborhood” at school-level and educational stage on depression. DSRS-C, Depression Self-Rating Scale for Children.

As for the school-level, “school trust and social cohesion” (β = –0.42, *p* < .05) and “perceived safety in school and neighborhood” (β = –0.73, *p* < .001) were negatively associated with depression. Furthermore, the significant cross-level interaction between educational stage and “perceived safety in school and neighborhood” at school-level (β = 0.55, *p* < .01) indicated that the relationship between safety at school-level and depression varied by the educational stage (elementary and junior high). The simple slopes analysis revealed that “perceived safety in school and neighborhood” at school-level was more strongly associated with depression among elementary students (*B* = –4.49, *p* < .001) than junior students (*B* = 0.57, *p* = .360) (Fig 2).

**Fig 3:**
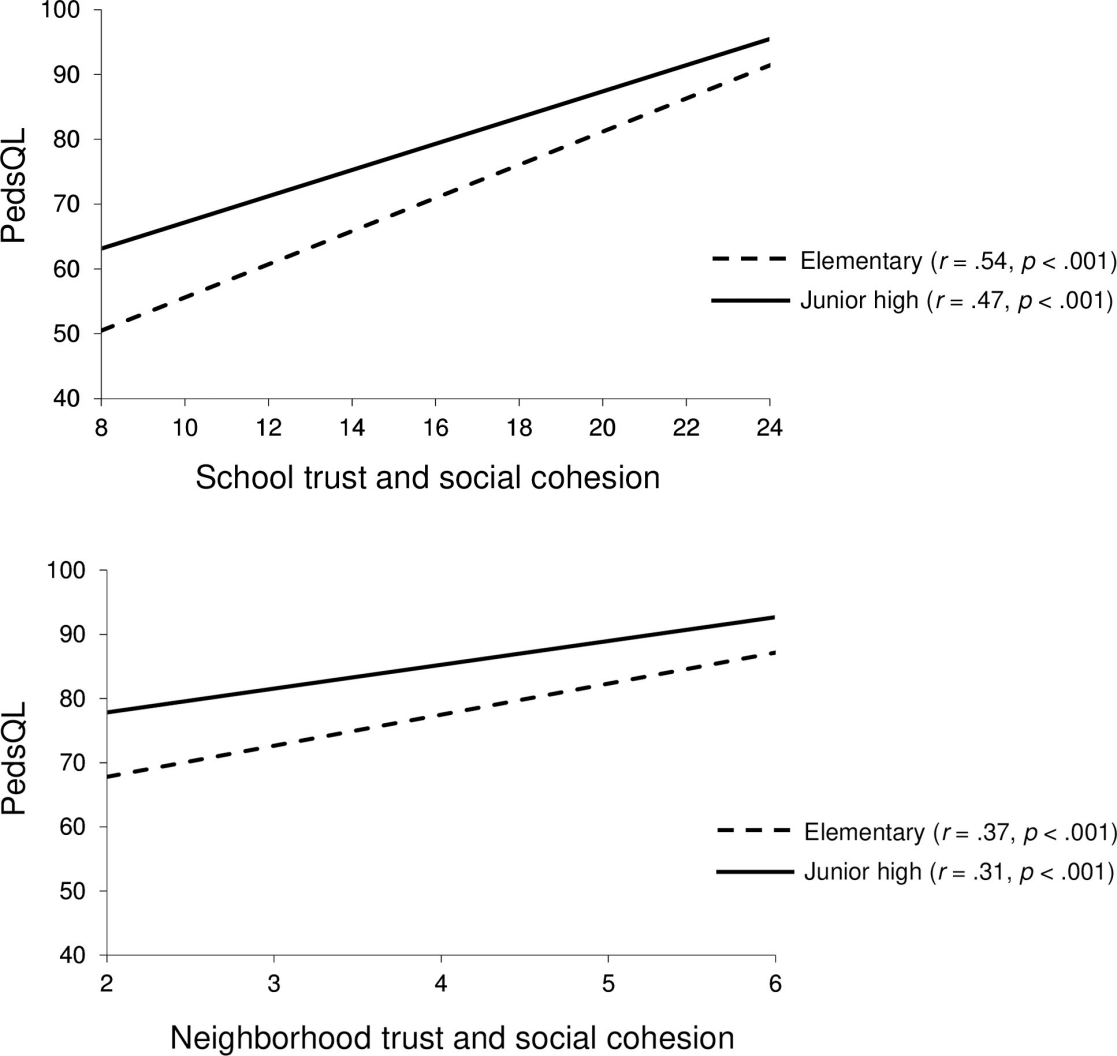
Interaction between social capital and educational stage on QoL. (a) Plots of the impacts of the interaction between “school trust and social cohesion” and educational stage on QoL. (b) Plots of the impacts of the interaction between “neighborhood trust and social cohesion” and educational stage on QoL.

Table 3 shows the results of the multilevel linear mixed-effect in which the criterion variable was QoL, measured by the PedsQL. Model 0 is an empty model without explanatory variables. The ICC was 0.07, which means that 7% of the observed individual differences in QoL can be attributed to the school-level. DEFF was 540.56, which means that this data has hierarchical structure and multilevel modeling is needed. Since DIC decreased with each model, Model 5 was adopted as the final model (individual-level: *R*^2^ = .26, *p* < .001, school-level: *R*^2^ = .49, *p* < .001). As regard with individual-level, the educational stage and all three SCQ-AS subscales exhibited a significant main effect on the PedsQL (QoL). The subscale “school trust and social cohesion” (β = 0.41, *p* < .001) showed the stronger relationship with QoL than other subscales (“perceived safety in school and neighborhood” (β = 0.15, *p* < .001) and “neighborhood trust and social cohesion” (β = 0.09, *p* < .001). Additionally, a significant interaction was observed between the educational stage and “school trust and social cohesion” (β = –0.02, *p* < .05), and also a significant interaction was observed between the educational stage and “neighborhood trust and social cohesion” (β = –0.02, *p* < .05). The simple slopes analysis revealed that “school trust and social cohesion” was more strongly associated with QoL among the elementary students (*B* = 2.03, *p* < .001) than the junior high students (*B* = 1.72, *p* < .001) (Fig 3A). Likewise, “neighborhood trust and social cohesion” was more strongly associated with QoL among the elementary students (*B* = 1.25, *p* < .001) than the junior high students (*B* = 0.66, *p* < .001) (Fig 3B). The higher the score on the subscale “neighborhood trust and social cohesion”, better QoL, especially among elementary students.

**Table 3:** Multilevel linear mixed effect model coefficients for PedsQL.

	Model 0	Model 1	Model 2	Model 3	Model 4	Model 5
	β (*SE*)	β (*SE*)	β (*SE*)	β (*SE*)	β (*SE*)	β (*SE*)
**Individual-level**						
Sex		0.02 (0.01) *	0.00 (0.01)	0.00 (0.01)	–0.00 (0.01)	0.00 (0.01)
Educational Stage		0.21 (0.03) ***	–0.73 (0.06) ***	0.69 (0.09) ***	0.67 (0.08) ***	7.21 (2.95) ***
SCQ-AS						
School trust and social cohesion			0.28 (0.02) ***	0.44 (0.02) ***	0.44 (0.02) ***	0.41 (0.02) ***
Perceived safety in school and neighborhood			0.10 (0.01) ***	0.16 (0.02) ***	0.16 (0.02) ***	0.15 (0.02) ***
Neighborhood trust and social cohesion			0.05 (0.01) ***	0.10 (0.02) ***	0.10 (0.02) ***	0.09 (0.02) ***
**Individual-level Interaction**						
Educational Stage × School trust and social cohesion				–0.28 (0.10) *	–0.28 (0.08) ***	–0.02 (0.01) *
Educational Stage × Perceived safety in school and neighborhood				–0.09 (0.06)	–0.09 (0.06) *	–0.01 (0.01)
Educational Stage × Neighborhood trust and social cohesion				–0.12 (0.05) *	–0.13 (0.05) *	–0.02 (0.01) *
**School-level**						
School size					0.25 (0.10) *	0.32 (0.13) *
SCQ-AS						
School trust and social cohesion					0.48 (0.18) ***	0.26 (0.16)
Perceived safety in school and neighborhood					0.37 (0.15) *	0.48 (0.15) **
Neighborhood trust and social cohesion					0.20 (0.20)	0.08 (0.19)
**Cross-level interaction (Individual × school-level)**						
Educational Stage × School trust and social cohesion						0.24 (0.26)
Educational Stage × Perceived safety in school and neighborhood						–0.43 (0.21)
Educational Stage × Neighborhood trust and social cohesion						–0.15 (0.26)
**Model Information criteria**						
Deviance (DIC)	61792.14	61791.00	57843.42	59319.02	59316.78	59059.61
**ICC**	0.07	0.03	0.68	0.05	0.01	0.02

* *p* < .05

** *p* < .01

*** *p* < .001.

PedsQL, Pediatric Quality of Life Inventory 4.0 Generic Core Scales; SCQ-AS, Social Capital Questionnaire for Adolescent Students; DIC, Deviance information criterion.

As for school-level, school size (β = 0.32, *p* < .05) and “perceived safety in school and neighborhood” (β = 0.48, *p* < .01) were positively associated with QoL. There were no statistically significant cross-level interactions between educational stage and social capital at school-level.

## Discussion

We examined the relationship between social capital at both individual- and school-level and depression and the relationship between social capital at both individual- and school-level and QoL in elementary and junior high students attending all public schools in a single municipality. The results indicated that higher scores on all three subscales (“school trust and social cohesion,” “perceived safety in school and neighborhood,” and “neighborhood trust and social cohesion”) were associated with lower levels of depression and higher QoL in the students. We also examined how the relationship between social capital and both depression and QoL differs among elementary and junior high students. We revealed that the relationship between social capital both depression and QoL varies among elementary and junior high students.

### Relationship between social capital at individual-level and both depression and QoL

Our results showed that students with higher levels of social capital tend to have less depression and better QoL (Tables 2, 3). The results are consistent with previous studies that investigated the relationship between social capital and depression [33, 35, 36] and between social capital and QoL [39]. Although little is known about the effects of school social capital on mental health in children and adolescence, our study revealed that school social capital was associated more strongly with depression and QoL than neighborhood social capital does (Tables 2, 3). This finding corroborates the studies that found—using one-item or two-item instruments with unverified validity—that school social capital is more strongly associated with subjective well-being [29, 67] and self-esteem [68] than neighborhood social capital does. As pointed out in previous studies [29–31], this indicates the importance of school social capital as a component of children’s social capital. As previous research suggested that individual level cognitive social capital is protective against mental health problems in adults [40], our finding implies that a higher level of social capital in children can prevent mental health problems from worsening.

Our results also indicated the importance of neighborhood social capital alongside school social capital. This finding corroborates existing reports that neighborhood social capital is inversely associated with depression and positively associated with QoL [37, 69]. Specifically, in a study conducted in Taiwan, Wang and Fowler observed that, in urban areas, plentiful social interactions with neighbors were associated with mental health benefits in young people [70]. In this study, the results were consistent with the findings in the urban area of Taiwan.

The educational stage was positively associated with QoL, indicating that junior high students had better QoL than elementary students. The difference in the measured QoL scores by different age groups were also reported in another study, where adolescents (13–18 years old) showed higher QoL than children (8–12 years old) [71]. Our finding may be attributed to the system in Japan that assigns more school counselors to junior high schools than to elementary schools [72, 73]. To support this, one study reported that students receiving more frequent guidance and interventions from school counselors felt safer in schools and had better QoL, regardless of the number of students in the school [74].

### Relationship between social capital at school-level and both depression and QoL

The results of this study showed that depression and QoL could be explained by school differences, but only slightly. However, because hierarchical structure of the data was observed and the purpose of this study was to clarify the relationship between social capital at school-level and both depression and QoL, multilevel mixed-effects analysis was conducted. We found that school social capital and safety at the school-level were associated with depression, as well as at the individual-level. In other words, this indicated that students enrolled in schools with higher school social capital and/or safety had lower severity of depression. These findings were consistent with those reported in a longitudinal study, where school environment, including relationships with students and teachers, and a sense of safety at both the individual and school levels, predicted depression in adolescents [75].

Neighborhood social capital at individual-level was negatively associated with depression; however, we did not find significant associations between school-level neighborhood social capital and depression. The study by Aslund et al. found that lower neighborhood social capital at both the individual- and neighborhood-levels was associated with a higher risk of depression; however, school social capital was not measured [69]. The inconsistent findings between our study and the above-referenced study may be due to the confounding effects of school social capital at individual- and school-level.

Regarding QoL, only school-level safety was significantly associated with QoL. This result is consistent with previous studies that found higher neighborhood safety was associated with better QoL [76] and well-being [77]. This finding in our study suggests that pediatric QoL is more associated with safety at school-level than with school and neighborhood social capital at school-level.

The present study showed that school size was positively associated with QoL. One previous study reported that school size was slightly negatively associated with life satisfaction [78]. On the other hand, review studies [79] have indicated little association between objective physical environment (e.g. school size) and student-level outcomes. These contradictory findings may be explained by confounding factors that influence the association between school size and student-level outcomes. School-level factors such as school size, sector, location, student composition, and neighborhood characteristics tend to be significantly interrelated [80], suggesting that school size can function as a facilitator or detractor of other organizational forms and practices, which in turn promotes well-being of students [81]. For example, a review of the school size effect presents that smaller schools are associated with greater school engagement, including connectedness, than larger schools [82]. Therefore, positive association between school size and QoL could be attributed to the confounding of school-level factors that were not measured in the present study.

### Inter-educational stage differences (among elementary and junior high students) in the relationship between social capital and both depression and QoL

Our results revealed that the relationship between social capital and both depression and QoL differed between elementary and junior high students. To the best of our knowledge, we are the first to report that this relationship differs between the two educational stage (elementary and junior high). The variation in outcomes for depression indicates that “school trust and social cohesion” at individual-level is more strongly associated with reduced depression among junior high students than elementary students (Fig 1). However, the variation in outcomes for QoL indicates that “school trust and social cohesion” at individual-level is more strongly associated with better QoL among elementary students than junior high students (Fig 3A). The difference in the degree of association between social capital and depression might be accounted for by the expression of depressive symptoms which varies according to the developmental stage [83–85]. Since early adolescents exhibit depressive mood and despair as the symptoms of major depressive disorder [85, 86], the score of “school trust and social cohesion” could be more strongly associated with the DSRS-C score which mainly contains items of depressive mood in junior high students (Fig 1). Conversely, in elementary students (Fig 3A), the score of “school trust and social cohesion” could be more strongly associated with the PedsQL score that includes the physical functioning subscale related to physical symptoms of depression because prepubescent children tend to display physical symptoms of major depression [83–85].

In multilevel mixed-effect analysis for depression, cross-level interactions between educational stage and safety at school-level was statistically significant, indicating that the effect of educational stage on depression varies among schools. “Perceived safety in school and neighborhood” at school-level is more strongly associated with depression among elementary students than junior high students (Fig 2). This finding may be explained by the differences in response to social environment at different developmental stages. For example, a meta-analysis study revealed that children aged below 12 years who were exposed to community violence reported more internalizing symptoms compared with adolescents aged 12 years and older [87]. Children may have lower ability to express their thoughts and feelings about community violence than adolescents. For children, this may lead to fewer cognitive coping strategies and fewer opportunities to receive support from adults [88–90]. In addition, the studies have reported that exposure to community violence increases with age, and thus, suggested that adolescents can be more likely to develop better coping skills, or become desensitized to violence over time [91, 92]. In other words, children may have more difficulty adapting to their environment due to their limited skills than adolescents, and as a result, they could be more susceptible to social environment influences. These differences in social development and experience suggest that safety at the school-level is more strongly associated with depression among elementary students.

The variation in outcomes for QoL indicates that “neighborhood trust and social cohesion” at individual-level is more strongly associated with better QoL among elementary students than among junior high students (Fig 3B). Regarding the existing research on this age-based variation in the relationship between neighborhood social capital and mental health, neighborhood social capital was associated with mental health among prepubescent children [93], while another study [46] found no such relationship among young adolescents and suggested that, whereas prepubescent children spend much of their time in their neighborhood, adolescents are more likely to spend their time at school and in club activities with peer groups, which makes them less affected by neighborhood social capital. As similar results may have been observed in the present study. On the other hand, in a study conducted on young people aged 11, 13, and 15, Morgan and Haglund [29] reported that among the domains of social capital (family, school, and neighborhood), only school social capital is associated with well-being regardless of age. The inconsistency in the extant literature might be attributable to discrepancies in the measures of neighborhood social capital.

### Limitations and outlook

One strength of this study is the use of data based on a large community sample with a high rate of participation, which provided a mostly unbiased sample of age cohorts. Another strength concerned the fact that, unlike preceding studies on social capital, the present study used an instrument with confirmed reliability and validity, making it possible to compare the findings with other survey data obtained using the same instrument. These strengths were used to identify social capital related to depression and QoL in school-aged children by including school-level social capital and interactions in the model, which were thought to be confounded in previous studies.

The study also had several limitations. First, due to its cross-sectional design, the study was unable to show causal relationships. Second, the SCQ-AS instrument was unable to capture the influence of family social capital, as this domain was removed from the questionnaire during the scale development. Parcel and Dufer demonstrated that child social adjustment is affected by combinations of safety in school and family composition [94]. Family structure, including mother education and family income, is also associated with children’s development [95, 96]. Thus, family social capital and structure should be considered to gain further insight into the relationship between social capital and young people’s mental health. Third, structural social capital was not assessed separately from cognitive social capital. Research in adult populations suggests that individual cognitive social capital is protective against mental health problems and individual structural social capital is not associated with mental health problems [40, 97]. However, recent research reported extracurricular participation is associated with mental disorder in adolescents [98]. Thus, we still consider it a limitation that the relative impacts of structural and cognitive social capital on mental health were not shown. Research on the relationship between social capital and problem behaviors, such as smoking and drinking, has demonstrated the importance of the type of organized activities in which young people participate and the frequency with which they participate [99, 100]. Thus, when examining the impact of cognitive social capital on mental health, it is important to consider the role of structural social capital. Finally, although our data came from a large community-based sample, the sample was limited to a particular region. Accordingly, one should consider the regional characteristics when attempting to extrapolate the results.

## Conclusion

Social capital at both individual- and school-level was associated with reduced depression and better QoL in young people. Of the social capital domains examined, school social capital at individual-level showed the strongest relationship with these outcomes, suggesting that school social capital may mitigate mental health problems among young people. In addition, the relationship between social capital and mental health differed among elementary and junior high students. School social capital at individual-level was more strongly associated with depression among junior high students than elementary students. Whereas, school and neighborhood social capital at individual-level was more strongly associated with QoL among elementary students than junior high students. Regarding school-level, safety was more strongly associated with depression among elementary students than junior high students. These results suggest that the degree of association of social capital domains differs in mental health problems among the educational stage.

## References

[1] World Health Organization. Adolescent mental health. 2020 Sep 28 [cited 2020 December 21]. In: World Health Organization [Internet]. Geneva: WHO 1948 -. [about 2 screens]. Available from: https://www.who.int/news-room/fact-sheets/detail/adolescent-mental-health.

[2] Rapport MD, Denney CB, Chung KM, Hustace K. Internalizing behavior problems and scholastic achievement in children: Cognitive and behavioral pathways as mediators of outcome. J Clin Child Psychol. 2001; 30(4): 536–551. doi: 10.1207/S15374424JCCP3004_10 11708241

[3] Kessler RC, Berglund P, Demler O, Jin R, Merikangas KR, Walters EE. Lifetime prevalence and age-of-onset distributions of DSM-IV disorders in the National Comorbidity Survey Replication. Arch Gen Psychiatry. 2005; 62(6): 593–602. doi: 10.1001/archpsyc.62.6.593 15939837

[4] Kim-Cohen J, Caspi A, Moffitt TE, Harrington H, Milne BJ, Poulton R. Prior juvenile diagnoses in adults with mental disorder: Developmental follow-back of a prospective-longitudinal cohort. Arch Gen Psychiatry. 2003; 60(7): 709–717. doi: 10.1001/archpsyc.60.7.709 12860775

[5] Ministry of Education Culture, Sports Science and Technology. Jidō seito no mondaikōdō futōkō tō seito sidōjō no syomondai ni kansuru chōsa [Survey on various issues related to student guidance, including problem behavior and school refusal]. The Ministry of Education, Culture, Sports, Science and Technology. [Internet] 2019 Oct 17. [Cited 2020 December 21]. Available from: https://www.mext.go.jp/content/1410392.pdf. Japanese.

[6] Ministry of Health, Labour and Welfare. Reiwa 2 nenjū ni okeru jisatsu no jōkyō [Suicides during 2020 in Japan]. The Ministry of Health, Labour and Welfare. [Internet] 2021 Mar [Cited 2021 December 8]. Available from: https://www.npa.go.jp/safetylife/seianki/jisatsu/R03/R02_jisatuno_joukyou.pdf. Japanese.

[7] Allen CW, Diamond-Myrsten S, Rollins LK. School Absenteeism in Children and Adolescents. Am Fam Physician. 2018; 98(12): 738–744. 30525360

[8] Hawton K, Casañas I Comabella C, Haw C, Saunders K. Risk factors for suicide in individuals with depression: A systematic review. J Affect Disord. 2013; 147(1–3): 17–28. doi: 10.1016/j.jad.2013.01.004 23411024

[9] Thapar A, Collishaw S, Pine DS, Thapar AK. Depression in adolescence. Lancet. 2012; 379(9820): 1056–1067. doi: 10.1016/S0140-6736(11)60871-4 22305766 PMC3488279

[10] Bourdieu P. The forms of capital. In: Richadson J, eds. The Handbook of Theory: Research for the Sociology of Education. New York: Greenwood Press; 1986. p.241–258.

[11] Coleman JS. Social capital in the creation of human capital. Am J Sociol. 1988; 94: S95–S120. doi: 10.1086/228943

[12] Putnam RD. Making democracy work. New Jersey: Princeton University Press; 1993.

[13] Putnam RD. Bowling alone: The collapse and revival of American community. New York: Simon and Schuster; 2000.

[14] Villalonga-Olives E, Kawachi I. The measurement of social capital. Gac Sanit. 2015; 29: 62–64. doi: 10.1016/j.gaceta.2014.09.006 25444390

[15] Rostila M. The facets of social capital. J Theory Soc Behav. 2011; 41: 308–326. doi: 10.1111/j.1468-5914.2010.00454.x

[16] Lin N. Social capital: A Theory of Social Structure and Action. Cambridge: Cambridge university press; 2002.

[17] Carpiano RM, Lisa MF. Questions of trust in health research on social capital: what aspects of personal network social capital do they measure? Soc Sci Med. 2014; 116: 225–234. doi: 10.1016/j.socscimed.2014.03.017 24721251

[18] Kawachi I. Commentary: social capital and health: making the connections one step at a time. Int J Epidemiol. 2006; 35(4): 989–993. doi: 10.1093/ije/dyl117 16870679

[19] Joye D, Sapin M, Wolf C. Measuring Social Networks and Social Resources: An Exploratory ISSP Survey around the World. Köln: GESIS—Leibniz-Institut für Sozialwissenschaften. 2019. [Cited 2021 September 6] Available from: https://nbn-resolving.org/urn:nbn:de:0168-ssoar-62256-9. doi: 10.21241/ssoar.62256

[20] Kawachi I, Berkman LF. Social cohesion, social capital, and health. In Berkman LF, Kawachi I, editors. Social epidemiology. New York: Oxford University Press; 2000, p.174–190.

[21] Islam MK, Merlo J, Kawachi I, Lindström M, Gerdtham UG. Social capital and health: Does egalitarianism matter? A literature review. Int J equity in health. 2006; 5(1): 1–28. doi: 10.1186/1475-9276-5-3 16597324 PMC1524772

[22] Kelly BD, Davoren M, Mhaoláin AN, Breen EG, Casey P. Social capital and suicide in 11 European countries: An ecological analysis. Soc Psychiatry Psychiatr Epidemiol. 2009; 44(11): 971–977. doi: 10.1007/s00127-009-0018-4 19277436

[23] Kennedy BP, Kawachi I, Prothrow-Stith D, Lochner K, Gupta V. Social capital, income inequality, and firearm violent crime. Soc Sci Med. 1998; 47(1): 7–17. doi: 10.1016/s0277-9536(98)00097-5 9683374

[24] Harpham T. The measurement of community social capital through surveys. In: Kawachi I, Subramanian S, Kim D, editors. Social Capital and Health. New York: Springer; 2008. pp. 51–62.

[25] Uphoff N. Understanding social capital: Learning from the analysis and experience of participation. In: Dasgupta P, Serageldin I, editors. Social Capital: A Multifaceted Perspective. Washington DC: The World Bank; 2000. pp. 215–252.

[26] Ferguson KM. Social capital and children’s wellbeing: A critical synthesis of the international social capital literature. Int J Soc Welf. 2006; 15(1): 2–18. doi: 10.1111/j.1468-2397.2006.00575.x

[27] McPherson KE, Kerr S, McGee E, Morgan A, Cheater FM, McLean J, et al. The association between social capital and mental health and behavioural problems in children and adolescents: an integrative systematic review. BMC Psychol. 2014; 26;2(1):7. doi: 10.1186/2050-7283-2-7 25566380 PMC4270040

[28] Vacchiano M, Bolano D. Leisure Activities, Social Capital and Mental Health: A Study Among Young Adults in Switzerland. Conference: American Sociological Association—ASA Annual Meeting; 2020 Aug8-11; San Francisco, US.

[29] Morgan A, Haglund BJ. Social capital does matter for adolescent health: Evidence from the English HBSC study. Health Promot Int. 2009; 24(4): 363–372. doi: 10.1093/heapro/dap028 19717401

[30] Takakura M. Does social trust at school affect students’ smoking and drinking behavior in Japan? Soc Sci Med. 2011; 72(2): 299–306. doi: 10.1016/j.socscimed.2010.11.003 21146276

[31] Weare K, Nind M. Mental health promotion and problem prevention in schools: What does the evidence say? Health Promot Int. 2011; 26 Suppl 1: i29–69. doi: 10.1093/heapro/dar075 22079935

[32] Sakai-Bizmark R, Richmond TK, Kawachi I, Elliott MN, Davies SL, Tortolero Emery S, et al. School social capital and tobacco experimentation among adolescents: Evidence from a cross-classified multilevel, longitudinal analysis. J Adolesc Health. 2020; 66(4): 431–438. doi: 10.1016/j.jadohealth.2019.10.022 32001140 PMC7089836

[33] McPherson K, Kerr S, McGee E, Cheater F, Morgan A. The role and impact of social capital on the health and wellbeing of children and adolescents: A systematic review. Glasgow Centre for Population Health. [Internet] 2013 Jan [Cited 2020 December 21] Available from: https://www.gcph.co.uk/assets/0000/3647/Social_capital_final_2013.pdf.

[34] Nielsen L, Koushede V, Vinther-Larsen M, Bendtsen P, Ersbøll AK, Due P, et al. Does school social capital modify socioeconomic inequality in mental health? A multi-level analysis in Danish schools. Soc Sci Med. 2015; 140: 35–43. doi: 10.1016/j.socscimed.2015.07.002 26189012

[35] Bosacki S, Dane A, Marini Z, Ylc‐Cura. Peer relationships and internalizing problems in adolescents: mediating role of self‐esteem. Emot Behav Diffic. 2007;12(4):261–82. doi: 10.1080/13632750701664293

[36] Ciairano S, Rabaglietti E, Roggero A, Bonino S, Beyers W. Patterns of adolescent friendships, psychological adjustment and antisocial behavior: The moderating role of family stress and friendship reciprocity. International Journal of Behavioral Development. 2007; 31(6): 539–48. doi: 10.1177/0165025407080573

[37] Glendinning A, West P. Young people’s mental health in context: Comparing life in the city and small communities in Siberia. Soc Sci Med. 2007; 65(6): 1180–1191. doi: 10.1016/j.socscimed.2007.05.012 17576031

[38] Birndorf S, Ryan S, Auinger P, Aten M. High self-esteem among adolescents: Longitudinal trends, sex differences, and protective factors. J Adolesc Health. 2005; 37(3): 194–201. doi: 10.1016/j.jadohealth.2004.08.012 16109338

[39] Drukker M, Kaplan C, Feron F, van Os J. Children’s health-related quality of life, neighbourhood socio-economic deprivation and social capital. A contextual analysis. Soc Sci Med. 2003; 57(5): 825–841. doi: 10.1016/s0277-9536(02)00453-7 12850109

[40] Ehsan AM, De Silva MJ. Social capital and common mental disorder: A systematic review. J Epidemiol Community Health. 2015; 69(10): 1021–1028. doi: 10.1136/jech-2015-205868 26179447

[41] Morrow V. Conceptualising social capital in reduction on the well-being of children and young people: A critical review. Sociol Rev, 1999; 47(4): 744–765. doi: 10.1111/1467-954X.00194

[42] Waterston T, Alperstein G, Stewart Brown S. Social capital: A key factor in child health inequalities. Arch Dis Child. 2004; 89(5): 456–459. doi: 10.1136/adc.2002.024422 15102639 PMC1719915

[43] De Silva MJ, Harpham T, Tuan T, Bartolini R, Penny ME, Huttly SR. Psychometric and cognitive validation of a social capital measurement tool in Peru and Vietnam. Soc Sci Med. 2006; 62(4): 941–953. doi: 10.1016/j.socscimed.2005.06.050 16095787

[44] Paiva PC, de Paiva HN, de Oliveira Filho PM, Lamounier JA, Ferreira e Ferreira E, Ferreira RC, et al. Development and validation of a social capital questionnaire for adolescent students (SCQ-AS). PLOS ONE. 2014; 9(8): e103785. doi: 10.1371/journal.pone.0103785 25093409 PMC4122396

[45] Saluja G, Iachan R, Scheidt PC, Overpeck MD, Sun W, Giedd JN. Prevalence of and risk factors for depressive symptoms among young adolescents. Arch Pediatr Adolesc Med. 2004; 158(8): 760–765. doi: 10.1001/archpedi.158.8.760 15289248

[46] Drukker M, Kaplan C, Schneiders J, Feron FJ, van Os J. The wider social environment and changes in self-reported quality of life in the transition from late childhood to early adolescence: a cohort study. BMC Public Health. 2006; 6: 133. doi: 10.1186/1471-2458-6-133 16707015 PMC1513203

[47] Ahlborg MG, Svedberg P, Nyholm M, Morgan A, Nygren JM. Into the realm of social capital for adolescents: A latent profile analysis. PLOS ONE. 2019; 14(2): e0212564. doi: 10.1371/journal.pone.0212564 30789947 PMC6383880

[48] Anderson LW., Jacobs J, Schramm S, Splittgerber F. School transitions: Beginning of the end or a new beginning? Int J Educ Res. 2000; 33(4): 325–339. doi: 10.1016/S0883-0355(00)00020-3

[49] Symonds JE, Galton M. Moving to the next school at age 10–14 years: An international review of psychological development at school transition. Rev Educ. 2014; 2(1): 1–27. doi: 10.1002/rev3.3021

[50] Rubin KH, Bowker JC, McDonald KL, Menzer, M. Peer relationships in childhood. In: Zelazo PD, editor. The Oxford Handbook of Developmental Psychology, Vol. 2: Self and Other. New York: Oxford University Press; 2013. pp. 242–275. doi: 10.1093/oxfordhb/9780199958474.013.0011

[51] Currie C, Zanotti C, Morgan A, Currie D, de Looze M, Roberts C, et al. Social determinants of health and well-being among young people. Health Behavior in School-aged Children (HBSC) study: International report from the 2009/2010 survey [Internet]. Copenhagen: WHO Regional Office for Europe; 2012 [Cited 2020 December 21]. Available from: https://www.euro.who.int/__data/assets/pdf_file/0003/163857/Social-determinants-of-health-and-well-being-among-young-people.pdf.

[52] Sampson RJ, Raudenbush SW, Earls F. Neighborhoods and violent crime: A multilevel study of collective efficacy. Science. 1997; 277: 918–924. doi: 10.1126/science.277.5328.918 9252316

[53] Takakura M, Hamabata Y, Ueji M, Kurihara A. Measurement of social capital at school and neighborhood among young people. School Health. 2014; 10: 1–8. doi: 10.20812/jash.SH-2014_067

[54] Hirota T, Adachi M, Takahashi M, Nakamura K. Cross-cultural adaptation and psychometric properties of the Social Capital Questionnaire for Adolescent Students among preadolescents and adolescents in Japan. Psychiatry Clin Neurosci. 2019; 73(9): 601–602. doi: 10.1111/pcn.12910 31271242

[55] Murata T, Shimizu A, Mori Y, Oshima S. Childhood depressive state in the school situation: Consideration from the Birleson’s Scale. The Japanese Journal of Psychiatry 1996; 1(2): 131–138. Japanese.

[56] Birleson P. The validity of depressive disorder in childhood and the development of a self-rating scale: A research report. J Child Psychol Psychiatry. 1981; 22(1): 73–88. doi: 10.1111/j.1469-7610.1981.tb00533.x 7451588

[57] Kobayashi K, Kamibeppu K. Measuring quality of life in Japanese children: Development of the Japanese version of PedsQL. Pediatr Int. 2010; 52(1): 80–88. doi: 10.1111/j.1442-200X.2009.02889.x 19496977

[58] Varni JW, Seid M, Rode CA. The PedsQL: Measurement model for the pediatric quality of life inventory. Med Care. 1999; 37(2): 126–139. doi: 10.1097/00005650-199902000-00003 10024117

[59] Heck RH, Thomas SL. An Introduction to Multilevel Modeling Techniques: MLM and SEM Approaches. 4th ed. New York: Routledge; 2020. doi: 10.4324/9780429060274

[60] Cyranowski JM, Frank E, Young E, Shear MK. Adolescent onset of the gender difference in lifetime rates of major depression: A theoretical model. Arch Gen Psychiatry. 2000; 57(1): 21–27. doi: 10.1001/archpsyc.57.1.21 10632229

[61] Wade TJ, Cairney J, Pevalin DJ. Emergence of gender differences in depression during adolescence: National panel results from three countries. J Am Acad Child Adolesc Psychiatry. 2002; 41(2):190–198. doi: 10.1097/00004583-200202000-00013 11837409

[62] Sommet N, Morselli D. Keep calm and learn multilevel linear modeling: A three-step procedure using SPSS, Stata, R, and MPlus. Int Rev Soc Psychol. 2021; 34(1): 24 doi: 10.5334/irsp.555

[63] Heisig JP, Schaeffer M. Why you should always include a random slope for the lower-level variable involved in a cross-level interaction. Eur Sociol Rev. 2019; 35(2), 258–279.

[64] Enders CK, Tofighi D. Centering predictor variables in cross-sectional multilevel models: a new look at an old issue. Psychol methods. 2007; 12(2): 121–138. doi: 10.1037/1082-989X.12.2.121 17563168

[65] Cain MK, Zhang Z. Fit for a Bayesian: An Evaluation of PPP and DIC for Structural Equation Modeling. Struct Equ Modeling: A Multidisciplinary Journal. 2019; 26(1): 39–50. doi: 10.1080/10705511.2018.1490648

[66] Muthén LK, Muthén BO. Mplus User’s Guide. Eighth Edition. Los Angeles, CA: Muthén & Muthén; 1998–2017.

[67] Eriksson U, Hochwälder J, Carlsund A, Sellström E. Health outcomes among Swedish children: The role of social capital in the family, school and neighbourhood. Acta Paediatr. 2012; 101(5): 513–517. doi: 10.1111/j.1651-2227.2011.02579.x 22211735

[68] Yugo M, Davidson MJ. Connectedness within social contexts: The relation to adolescent health. Healthc Policy. 2007; 2(3): 47–55. doi: 10.12927/hcpol.2007.18701 19305718 PMC2585451

[69] Aslund C, Starrin B, Nilsson KW. Social capital in relation to depression, musculoskeletal pain, and psychosomatic symptoms: A cross-sectional study of a large population-based cohort of Swedish adolescents. BMC Public Health. 2010; 10:715. doi: 10.1186/1471-2458-10-715 21092130 PMC3091587

[70] Wang SC, Fowler PJ. Social cohesion, neighborhood collective efficacy, and adolescent subjective well-being in urban and rural Taiwan. Am J Community Psychol. 2019; 63(3–4): 499–510. doi: 10.1002/ajcp.12324 30861156

[71] Varni JW, Burwinkle TM, Seid M. The PedsQL^TM^ 4.0 as a school population health measure: feasibility, reliability, and validity. Qual Life Res. 2006; 15: 203–215. doi: 10.1007/s11136-005-1388-z 16468077

[72] Ministry of Education, Culture, Sports, Science and Technology. School Health Statistics Research. The Ministry of Education, Culture, Sports, Science and Technology. [Internet] 2020 Mar 23. [Cited 2021 September 10]. Available from https://www.e-stat.go.jp/dbview?sid=0003146561.

[73] Ministry of Education, Culture, Sports, Science and Technology. Gakkō ni okeru kyōikusōdan ni kansuru siryō [Documents on educational consultation in schools]. The Ministry of Education, Culture, Sports, Science and Technology. 2017 Dec 17. [Cited 2021 September 10]. Available from https://www.mext.go.jp/b_menu/shingi/chousa/shotou/120/gijiroku/__icsFiles/afieldfile/2016/02/12/1366025_07_1.pdf. Japanese.

[74] Lapan RT, Gysbers NC, Sun Y. The impact of more fully implemented guidance programs on the school experiences of high school students: A statewide evaluation study. J Couns Dev. 1997; 75(4): 292–302. doi: 10.1002/j.1556-6676.1997.tb02344.x

[75] Brière FN, Pascal S, Dupéré V, Janosz M. School environment and adolescent depressive symptoms: a multilevel longitudinal study. Pediatrics. 2013; 131(3): e702–e708. doi: 10.1542/peds.2012-2172 23400608

[76] Martin G, Graat M, Medeiros A, Clark AF, Button BLG, Ferguson KN, et al. Perceived neighbourhood safety moderates the relationship between active school travel and health-related quality of life. Health Place. 2021;70:102623. doi: 10.1016/j.healthplace.2021.102623 34265633

[77] Aminzadeh K, Denny S, Utter J, Milfont TL, Ameratunga S, Teevale T, et al. Neighbourhood social capital and adolescent self-reported wellbeing in New Zealand: a multilevel analysis. Soc Sci Med. 2013; 84:13–21. doi: 10.1016/j.socscimed.2013.02.012 23517699

[78] Cho E YN. A multilevel analysis of life satisfaction among secondary school students: Do school-level factors matter? Child Youth Serv Rev. 2019; 102: 231–242. doi: 10.1016/j.childyouth.2019.05.002

[79] Milfont TL, Denny SJ. Everyday Environments and Quality of Life: Positive School and Neighborhood Environments Influence the Health and Well-Being of Adolescents. In: Fleury-Bahi G., Pol E., Navarro O, editors. Handbook of Environmental Psychology and Quality of Life Research. International Handbooks of Quality-of-Life. Springer: Cham; 2017. doi: 10.1007/978-3-319-31416-7_20

[80] McMillen BJ. School Size, Achievement, and Achievement Gaps. Educ Policy Anal Arch. 2004; 12: 58. doi: 10.14507/epaa.v12n58.2004

[81] Lee VE. School Size and the Organization of Secondary Schools. In: Hallinan MT, editor. Handbook of the Sociology of Education. Handbooks of Sociology and Social Research. Springer: Boston, MA; 2000. doi: 10.1007/0-387-36424-2_15

[82] Leithwood K, Jantzi D. A review of empirical evidence about school size effects: A policy perspective. Rev Edu Res. 2009; 79(1): 464–490. doi: 10.3102/0034654308326158

[83] Fox C, Buchanan-Barrow E, Barrett M. Children’s conceptions of mental illness: A naïve theory approach. Br J Dev Psychol. 2010; 28(Pt 3): 603–625. doi: 10.1348/026151009x461366 20849036

[84] Korczak DJ, Ofner M, LeBlanc J, Wong S, Feldman M, Parkin PC. Major depressive disorder among preadolescent Canadian children: Rare disorder or rarely detected? Acad Pediatr. 2017; 17(2): 191–197. doi: 10.1016/j.acap.2016.10.011 27989927

[85] Georgakakou-Koutsonikou N, Williams JM. Children and young people’s conceptualizations of depression: A systematic review and narrative meta-synthesis. Child Care Health Dev. 2017; 43(2): 161–181. doi: 10.1111/cch.12439 28090667

[86] Tripković I, Roje R, Krnić S, Nazor M, Karin Ž, Čapkun V. Depression and self-esteem in early adolescence. Cent Eur J Public Health. 2015; 23(2): 166–169. doi: 10.21101/cejph.a4017 26851429

[87] Fowler PJ, Tompsett CJ, Braciszewski JM, Jacques-Tiura AJ, Baltes BB. Community violence: A meta-analysis on the effect of exposure and mental health outcomes of children and adolescents. Dev Psychopathol. 2009; 21: 227–59. doi: 10.1017/S0954579409000145 19144232

[88] Compas BE. Coping with stress during childhood and adolescence. Psychol Bull. 1987; 101(3): 393–403. doi: 10.1037/0033-2909.101.3.393 3602247

[89] Farver JAM, Xu Y, Eppe S, Fernandez A, Schwartz D. Community violence, family conflict, and preschoolers’ socioemotional functioning. Dev Psychol. 2005; 41(1): 160–170. doi: 10.1037/0012-1649.41.1.160 15656746

[90] Grant KE, Compas BE, Thurm AE, McMahon SD, Gipson PY, Campbell AJ, et al. Stressors and child and adolescent psychopathology: Evidence of moderating and mediating effects. Clin Psychol Rev. 2006; 26(3): 257–283. doi: 10.1016/j.cpr.2005.06.011 16364522

[91] Fitzpatrick KM. Exposure to violence and presence of depression among low-income, African-American youth. J Consult Clin Psychol. 1993; 61(3): 528–531. doi: 10.1037//0022-006x.61.3.528 8326056

[92] Rasmussen A, Aber MS, Bhana A. Adolescent coping and neighborhood violence: perceptions, exposure, and urban youths’ efforts to deal with danger. Am J Community Psychol. 2004; 33(1–2): 61–75. doi: 10.1023/b:ajcp.0000014319.32655.66 15055755

[93] Xue Y, Leventhal T, Brooks-Gunn J, Earls FJ. Neighborhood residence and mental health problems of 5- to 11-year-olds. Arch Gen Psychiatry. 2005; 62(5): 554–563. doi: 10.1001/archpsyc.62.5.554 15867109

[94] Parcel TL, Dufur MJ. Capital at home and at school: Effects on child social adjustment. J Marriage Fam. 2001; 63(1): 32–47. doi: 10.1111/j.1741-3737.2001.00032.x

[95] Carlson MJ, Corcoran ME. Family structure and children’s behavioral and cognitive outcomes. J Marriage Fam. 2001; 63(3): 779–792. doi: 10.1111/j.1741-3737.2001.00779.x

[96] Violato M, Petrou S, Gray R, Redshaw M. Family income and child cognitive and behavioural development in the United Kingdom: does money matter? Health Econ. 2011; 20(10): 1201–1225 doi: 10.1002/hec.1665 20945340

[97] De Silva MJ, McKenzie K, Harpham T, Huttly SR. Social capital and mental illness: A systematic review. J Epidemiol Community Health. 2005; 59(8): 619–627. doi: 10.1136/jech.2004.029678 16020636 PMC1733100

[98] Hirota T, Paksarian D, He JP, Inoue S, Stapp EK, Van Meter A, et al. Associations of Social Capital with Mental Disorder Prevalence, Severity, and Comorbidity among U.S. Adolescents. J Clin Child Adolesc Psychol. 2021 Mar 3:1–12. doi: 10.1080/15374416.2021.1875326 33656940 PMC8413396

[99] Takakura M. Relations of participation in organized activities to smoking and drinking among Japanese youth: Contextual effects of structural social capital in high school. Int J Public Health. 2015; 60(6): 679–689. doi: 10.1007/s00038-015-0697-4 26123654

[100] Zambon A, Morgan A, Vereecken C, Colombini S, Boyce W, Mazur J, et al. The contribution of club participation to adolescent health: Evidence from six countries. J Epidemiol Community Health. 2010; 64(1): 89–95. doi: 10.1136/jech.2009.088443 20007634

